# Facile Synthesis of Sustainable Biomass-Derived Porous Biochars as Promising Electrode Materials for High-Performance Supercapacitor Applications

**DOI:** 10.3390/nano12050866

**Published:** 2022-03-04

**Authors:** Ravi Moreno Araujo Pinheiro Lima, Glaydson Simões dos Reis, Mikael Thyrel, Jose Jarib Alcaraz-Espinoza, Sylvia H. Larsson, Helinando Pequeno de Oliveira

**Affiliations:** 1 Institute of Materials Science, Federal University of Sao Francisco Valley, Petrolina 56304-205, Brazil; raviplima.engmec@gmail.com (R.M.A.P.L.); helinando.oliveira@univasf.edu.br (H.P.d.O.); 2 Department of Forest Biomaterials and Technology, Swedish University of Agricultural Sciences, Biomass Technology Centre, SE-90183 Umeå, Sweden; mikael.thyrel@slu.se (M.T.); sylvia.larsson@slu.se (S.H.L.); 3 Departamento de Química, Universidad Autónoma Metropolitana, Iztapalapa, Mexico City 09340, Mexico; josejarib@gmail.com

**Keywords:** spruce bark electrodes, spruce bark-supercapacitors, impedance, areal capacitance, electrical double layer capacitance

## Abstract

Preparing sustainable and highly efficient biochars as electrodes remains a challenge for building green energy storage devices. In this study, efficient carbon electrodes for supercapacitors were prepared via a facile and sustainable single-step pyrolysis method using spruce bark as a biomass precursor. Herein, biochars activated by KOH and ZnCl_2_ are explored as templates to be applied to prepare electrodes for supercapacitors. The physical and chemical properties of biochars for application as supercapacitors electrodes were strongly affected by factors such as the nature of the activators and the meso/microporosity, which is a critical condition that affects the internal resistance and diffusive conditions for the charge accumulation process in a real supercapacitor. Results confirmed a lower internal resistance and higher phase angle for devices prepared with ZnCl_2_ in association with a higher mesoporosity degree and distribution of Zn residues into the matrix. The ZnCl_2_-activated biochar electrodes’ areal capacitance reached values of 342 mF cm^−2^ due to the interaction of electrical double-layer capacitance/pseudocapacitance mechanisms in a matrix that favors hydrophilic interactions and the permeation of electrolytes into the pores. The results obtained in this work strongly suggest that the spruce bark can be considered a high-efficiency precursor for biobased electrode preparation to be employed in SCs.

## 1. Introduction

The development of sustainable and efficient energy storage systems (ESS) attracts massive attention in the literature due to their wide range of applications, from portable electronic devices to hybrid electric vehicles [1,2,3]. Among different types of ESS, super-capacitors (SCs) have favorable properties of relatively high specific power density (10 to 100,000 W/kg), outstanding cycling stability (minimal change in the electrochemical response after a few thousands of reuses), low resistance, fast charge/discharge, and a wide range of applications [4,5]. There are two typical mechanisms for charge storage in supercapacitors: electric double-layer capacitance (EDLCs) and pseudocapacitance (PC) [3,4]. EDLCs make use of the diffusion and accumulation of double-layer charges formed by the adsorption of electrolyte ions on the electrode’s surface (physisorption); thus, electrodes with very high specific surface area (SSA) and a high level of hydrophilicity are generally required to fabricate EDLCs [3,4]. Differently, pseudocapacitors store energy not only through the formation of an EDL but also through reversible redox reactions with the fast insertion of the electrolyte ions onto the surface layer of the electrode [3,4].

The combination of materials with the prevailing pseudocapacitive response and one second class of materials with properties of EDLC are extensively explored to create high-performance electrodes for SCs. A critical material for the fabrication of SCs is graphite, typically employed as an electrode material [6,7,8,9]. However, graphite mining and processing are costly and have a sizeable CO_2_ footprint [6,7], and graphene and carbon nanotubes synthesis processes are complex and expensive.

Besides, the theoretical capacity of graphite is limited to 372 mAh g^−1^ with the average area normalized capacitance of graphite being 15 µF cm^−2^ [10], and very low SSA (usually, natural graphite has SSA below 10 m^2^ g^−1^ ). However, after treatments, graphite can reach SSA values much higher (500 m^2^ g^−1^). In the case of graphene, it has a theoretical capacitance of up to 550 F g^−1^ based on the theoretical surface area (2630 m^2^ g^−1^) [11].

Therefore, it is important to study new ways of replacing these high-cost and complex materials for cheaper and sustainable ones [2,3,12,13,14]. Extensive research is being conducted to develop innovative concepts using biomass materials as precursors to develop biochar-based materials employed as electrodes for SC [2,3,14]. Biochars can be made from any biomass and have an adaptable structure that, through different pre-treatments, can be modified to reach a high SSA, even higher than 3300 m^2^ g^−1^ [12], well-developed porosity with varying pore sizes, and more oxygen and nitrogen surface functional groups on its surfaces, making them very suitable to be used as electrodes for SCs [3,15,16]. In addition, using biochars is both environmentally friendly and economically advantageous. Biochar can be made through simultaneous pyrolysis and chemical activation, and by optimizing the pyrolysis and activation conditions, the biochar can be designed for specific applications [15,16], but the properties of the original feedstock are nevertheless decisive for the structure and functionality of the obtained material [3,15,16].

The biochar can be tailored to obtain properties that provide high-performance SCs electrodes by optimizing the production method. For instance, the two most common chemical activations, by zinc chloride (ZnCl_2_) and potassium hydroxide (KOH), yield biochars with distinctly different properties [2,3,14]; the literature shows that ZnCl_2_, usually, produces biochars with more developed mesoporosity while biochars activated with KOH are mostly microporous structures [17]. This difference is fairly important in choosing the method and activation agent for the biochars preparation with tailored properties. The activation mechanism of KOH is based on a reaction with carbon, with the CO and H_2_ considered as subproducts, while the interaction mechanism with ZnCl_2_ is based on catalytic dehydration with the ZnCl_2_ acting as a skeleton during carbonization, with positive effects on pore structure and specific surface area. The activation incorporates graphite nitrogen and pyridine nitrogen, resulting in the enhancement of the oxygen adsorption/ reduction, with direct consequences on the electron transfer. In particular, high pyridine-nitrogen content is an important requisite to reach adequate conditions in terms of good mesoporosity and pseudocapacitive contribution [13].

Basic research is required to optimize the production of electrodes adapted to the size of the electrolyte ions, thereby providing high conductivity and good physio-chemical stability that improve the electrochemical performance of biochar-based SCs (e.g., lifetime, capacity, and safety) [2,3,13,14].

Jiang et al. [18] used lignin-rich biomass for making SC anodes. The SCs exhibited a specific capacitance of 80.9 and 92.7 F g^−1^ at the constant current density of 100 mA g^−1^. He et al. [19] used peanut shells as a precursor to producing carbon electrodes for SCs. Carbon electrodes (CEs) were created through chemical activation with ZnCl_2_. The fabricated SCs showed a specific capacitance of 99 F g^−1^ and high energy density (19.3 Wh kg^−1^) at a high-power density of 1007 W kg^−1^. Wu et al. [20] prepared microporous carbon materials from almond nutshells by KOH and HNO_3_ activation. The carbon materials displayed SSA values of 1363 and 327.7 m^2^ g^−1^ for KOH and HNO_3_, respectively, while their respective specific capacitances were 272.3 and 286.1 F g^−1^ at 1 A g^−1^.

Although some biomass precursors are employed in electrodes for SCs, there is still a lot to understand about using biomass carbon materials as electrodes for SCs. It is well-known that the structure of the original biomass severely restricts the final structure of the synthesized carbon material. However, there is a lack of understanding regarding the relationship between biomass properties, process conditions, the resulting biochar properties, and the electrochemical performance of the carbon electrodes in SCs applications.

Generally, softwood bark is a low-value residue in forest–industrial production chains. In this work, Norway spruce (*Picea abies* (Karst.) L.) bark was used as the main precursor for producing electrode biochar for SCs. The bark’s constitution of cellulose, hemicellulose, and lignin [16], makes it a suitable precursor for biochar to be used as electrodes for SCs [2,3,14]. Herein, we describe the preparation of two different electrode types, made from KOH- and ZnCl_2_-activated Norway spruce bark, respectively. These two electrodes were fully characterized by various physicochemical and electrochemical analyses, displaying high specific capacitance and good rate capability.

To the best of our knowledge, only one paper employed spruce bark as the main precursor for the preparation of biochar electrodes for SCs [11]. Besides, the effect of different chemical activation and responses that describe the structural and chemical properties of the biochar materials and their resulting electrode performance are fully evaluated. These two electrodes were fully characterized by various physiochemical and electrochemical analyses, displaying high specific capacitance and good rate capability.

## 2. Materials and Methods

### 2.1. Chemicals and Reagents

The Norway spruce bark was provided by a Holmen paper (Stockholm, Sweden) and pulp mill in North-Eastern Sweden. The spruce bark was dried and milled (Fritsch Pulverisette 14, Idar-Oberstein, Germany) at a screen size of 500 µm. The chemicals potassium hydroxide (KOH), polyvinylidene fluoride (PVDF), zinc chloride (ZnCl_2_), and hydroxide chloride (HCl) were purchased from Sigma-Aldrich (St. Louis, MO, USA). Dimethylformamide (DMF) was purchased from Dinamica (Gorizia, Italy), and the carbon black was purchased from Micromeritics (Norcross, GA, USA).

### 2.2. Preparation Process

The biochars were prepared as follows: first, 50.0 g of bark were mixed with KOH at a ratio of 1:1 (weight), and then mixed with 30 mL of distilled water in a melting pot until a homogeneous paste was obtained [21,22]. The same procedure was followed with the ZnCl_2_ activation process. The mixtures were left for 2 h at ambient temperature, whereafter it was dried in a drying oven at 105 °C overnight. Pyrolysis was performed at 900 °C for 2 h under nitrogen flow, with an initial heating rate of 10 °C per min. After pyrolysis, the samples were milled and washed with 1.0 M and 6.0 HCl for KOH and ZnCl_2_ biochars, respectively. Finally, a washing step with distilled water was performed several times until the pH value of the filtrate reached a neutral value [21,22].

#### 2.2.1. Biochar Characterization

The SSA and porosity data of the biochars were evaluated via nitrogen adsorption–desorption isotherms using a Tristar 3000 apparatus, Micrometrics Instrument Corp. The biochars were subjected to degasification at 180 °C for 3 h, and the SSA and pore size distribution were obtained using the Brunauer–Emmett–Teller (BET) method.

The morphology of biochars was evaluated from the scanning electron microscopy (SEM) technique, using a Zeiss-Gemini microscope, and images were made at 20 µm scales with 2.5k× of magnification.

XPS analysis of the biochars was collected using a Kratos Axis Ultra DLD electron spectrometer using a monochromated Al K_α_ source operated at 150 W. An analyzer of 160 eV for acquiring survey spectra and 20 eV for individual photoelectron lines were used. The samples were gently hand-pressed using a clean Ni spatula into the powder sample holder. Due to the electrical conductive behavior of the carbonaceous material, no charge neutralization system was used. The binding energy (BE) scale was calibrated following the ASTM E2108 and ISO 15472 standards. Processing of the spectra was accomplished with the Kratos software.

Raman spectra were collected using a Bruker Bravo spectrometer (Bruker, Ettlingen, Germany) connected to a docking measuring station. Shortly, 0.5 g of each biochar powder was placed in 2.5 mL glass vials and scanned in the 300–3200 cm^−1^ spectral range at 4 cm^−1^ resolution for 256 scans. Min–Max normalization over the 1000–2000 cm^−1^ region and smoothing (9 points) was conducted using the built-in functions of the OPUS software (version 7, Bruker Optik GmbH, Ettlingen, Germany). No baseline correction was needed.

Fourier transform infrared spectroscopy (FTIR) was exploited to determine the functional groups of the biochars. The FTIR spectra were recorded over the wavenumber range of 4000–400 cm^−1^, utilizing a Bruker IFS 66v/S instrument (Bruker Optics, Ettlingen, Germany) with an acquisition of 64 scans min^−1^ and resolution of 4 cm^−1^.

The X-ray diffraction pattern was measured in a Rigaku Miniflex X-ray diffractometer (Rigaku Corporation, Tokyo, Japan) with CuKα (λ = 1.4518 Å), with operation parameters of 40 kV and a current of 15 mA in a continuous can from 5° to 6° with a step of 0.02°, speed of 10°/min.

#### 2.2.2. Preparation of Powder and Assembly of Two-Electrode Supercapacitors

**Figure 1:**
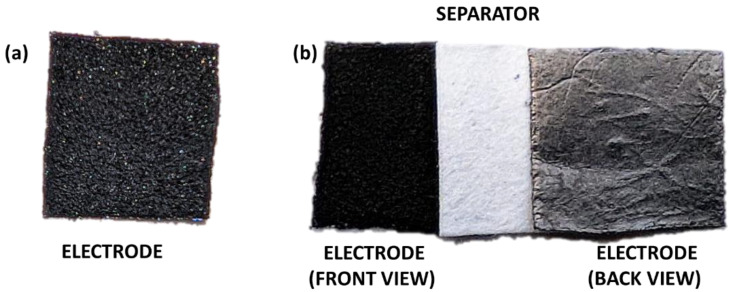
(**a**) Frontal view of the electrode and (**b**) disposition of components (symmetric electrodes and separator) in an assembled device.

As a first step in the electrode preparation, a slurry containing biomass charcoal (KOH biochar, ZnCl_2_ biochar), carbon black, and PVDF in a mass ratio of 0.70, 0.20, and 0.10, respectively, was created. Firstly, 10 mg of PVDF was added to 500 µL of DMF; then, the solution was stirred for 5 min at 85 °C to solubilize the polymer. After that, 70 mg of biomass charcoal and 20 mg of carbon black were added. The resulting solution was kept under stirring for 10 min at 85 °C. After that, 1 cm × 1 cm graphite electrodes were applied as support (current collector to be coated with slurry). The electrode was prepared by dropping 25 µL of the as-prepared slurry on the surface of the graphite paper. After evaporating the solvent, the coated graphite paper was placed on a hotplate at 70 °C for 5 min. This process was repeated until the surface of the graphite paper was completely covered. At the end of this process, two electrode types were obtained, one containing the KOH biochar and the other the ZnCl_2_ biochar. The SC was assembled in a sandwich-type, where a filter paper separated the electrodes. Both electrodes and the separator were impregnated with a KOH 5 M solution. Pictures for the assembled device are shown in Figure 1a,b, in which it is possible to identify the electrodes, the separator, and the final disposition of the parts.

## 3. Results and Discussion

### 3.1. Textural Properties and Morphology of the Biochars

**Figure 2:**
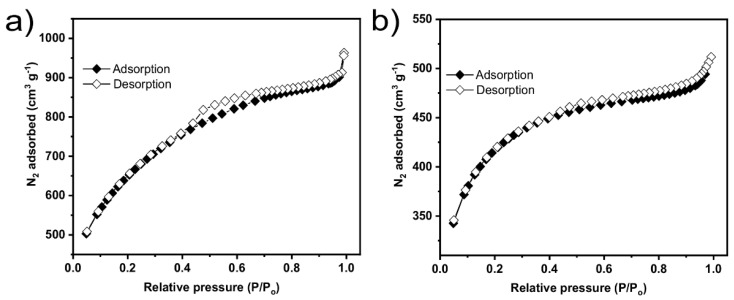
Nitrogen isotherms of the biochars (**a**) KOH−biochar and (**b**) ZnCl_2_−biochar.

SSA and porosity of the biobased carbon materials are essential properties that strongly influence their performance as materials regardless of their applications [2,3]. Both chemical activation methods yielded biochars with N_2_ adsorption/desorption isotherm curves close to type I as the nitrogen adsorption increased at low partial pressure (Figure 2). This describes an adsorption process resulting in micropores filling. However, hysteresis was observed in both samples, which characterizes mesoporous materials [23,24]. Both materials contain micro and mesoporosity due to the high N_2_ adsorption volume at low and high pressure [23,24]. To a large extent, the activation process influenced the amount of adsorbed N_2_, which also is reflected in the SSA values.

KOH biochar adsorbed a much higher N_2_ volume and, as a consequence, exhibited a much higher SSA value (2209 m^2^ g^−1^) (see Table 1). Since the pyrolysis conditions were the same for both biochars, the differences in their SSA values could be exclusively related to the chemical activation mechanisms based on KOH and ZnCl_2_. The reaction between KOH and biomass usually leads to a much more violent chemic during the thermal treatment, indicated by the lower yield (13.7% for KOH and 28.1% for ZnCl_2_).

KOH activation consists of several steps during the pyrolysis process-based solid–liquid reactions because potassium hydroxide is diluted in water. Above 700 °C, potassium metallic is formed and may enhance the porosity; the metallic ion K^+^ may act as a catalyst for gasification reactions, which helps to form and develop pore structures [25,26]. Additionally, a K intercalation process can occur; K ions may go between graphene layers of the biochar, widening its pore network and straining the structure, which potentializes the pore formation and reaches very high SSA values.

Activation with ZnCl_2_, which is a Lewis acid, is a potent dehydrating reagent as it catalyzes the decomposition of lignocellulosic compounds. This activation involves dehydration, depolymerization, and ring-opening [25,26,27,28]. ZnCl_2_ is an efficient catalyst for C-O and C-C bonds scission. Moreover, during pyrolysis, ZnCl_2_ starts to melt at 290 °C and may, if evenly mixed with the biomass, reach the biomass’ interior. Increasing the pyrolysis temperature leads to thermal dehydration of the zinc oxide chloride hydrate that forms a gaseous phase of ZnCl_2_ and a solid phase of zinc oxide. The gaseous phase of ZnCl_2_ is diffused through the carbonaceous structure to develop the pore network [26,27].

Thus, it can be stated that the activation process using KOH produced biochar with more developed porosity. In addition, the contributions of micropores and mesopores reaction in the biochar structures were evaluated. The percentage of mesopores in KOH biochar was 22.6% and in ZnCl_2_-BBC was 46%, while the share of micropores was 77.4% in KOH biochar and 54% in ZnCl_2_ biochar.

In ESS applications, an adequate combination of micro/mesopores distribution and electrolyte ions type and size is highly required because the electrolyte ions can be efficiently transported in small-sized pores, reaching high charge storage capability at low current density [2,3,29]. Miao et al. [30] reported that a large number of micropores (size between 1 and 2 nm) could facilitate the charge separation (due to the available sites for charge accumulation), which affects the overall energy density of the device.

**Figure 3:**
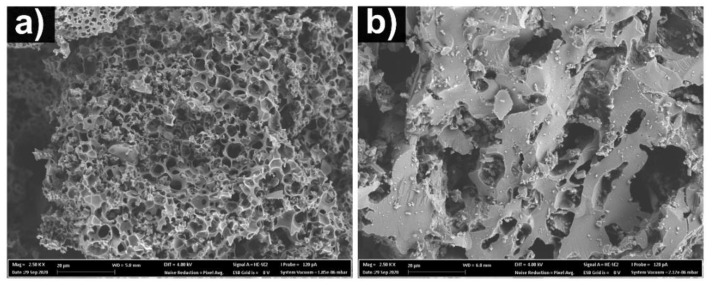
SEM images of AC the biochar samples (**a**) KOH and (**b**) ZnCl_2_, at 2.5k× of magnification.

SEM analysis was performed to examine the effect of the chemical activation on the surface morphology of the biochars. Figure 3 display the surface morphology of the two biochars. The images show that KOH activation results in a sponge-like structure (Figure 3a) full of roughness and irregular structure and tons of small holes. On the other hand, the sample made with ZnCl_2_ (Figure 3b) shows a much denser structure, with more elongated cavities and holes of different sizes and shapes.

Thus, by the SEM analysis (and to corroborate the BET analysis), it is possible to infer that the choice of activation method strongly affected the surface characteristics of the biochars. Moreover, both materials have a significant presence of macropores and ultra-macropores; vital because they serve as vectors to the electrolyte passage until it attains smaller pores (in the interior of the biochars). In terms of relevance for supercapacitor-based applications, it provides the possibility to provide electrolyte permeation along with the porous structure for the following step of charge accumulation into the cavities (more accessible channels).

### 3.2. Chemical and Functional Characterization of the Biochars

**Figure 4:**
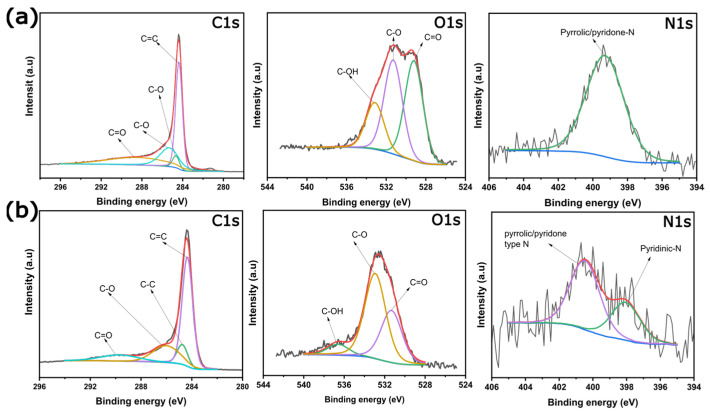
Carbon, oxygen, and nitrogen XPS spectra for (**a**) KOH biochar and (**b**) ZnCl_2_ biochar.

The chemical state and the main composition of the elements in the biochars were evaluated via the XPS technique [16,25,31], which gives detailed and valuable information about the effect of the chemical activation methods (KOH and ZnCl_2_) on the surface properties of the biochars. Figure 4a (KOH) and b (ZnCl_2_) show C 1s, O 1s, and N 1s spectra, carbon, oxygen, and nitrogen bonds, respectively.

The asymmetric C 1s spectra could be deconvoluted into four peaks as highlighted in Figure 4. These peaks correspond to C=C, C–C, C–O–C, and C=O bonds, typically of activated carbons [16,25,27].

O1s spectra exhibited some differences in their peak intensities regarding the chemical activation process. Both biochars present O1s spectra deconvoluted to three chemical oxygen states corresponding to (i) oxygen double-bonded with carbon (C=O) in carbonyl and quinone-like structures, (ii) oxygen singly bonded to carbon (C-O) in aromatic rings, in phenols and ethers, and (iii) hydroxyl groups (-OH). The presence of this oxygen species can improve the hydrophilicity degree of the sample, which can reflect in a better interaction between the solid and liquid phase (electrode–electrolyte), characterizing an advantage for permeation of aqueous electrolyte ions into the biochar-based electrode structure.

Figure 4 also show the deconvoluted N 1s spectra for the biochars; a single peak is observed in KOH biochar, which is related to pyrrolic nitrogen (400 eV) [14,16], while for ZnCl_2_ biochar, two peaks are observed, one at 397.9 eV which corresponds to pyridinic species [14,16] and at 400.5 eV that is related to pyrrolic nitrogen [14,16]. The presence of more N species in the ZnCl_2_ biochar can improve its electrochemical performances as an electrode for SCs. Nitrogen can act as an electron donor, enhancing the charge exchange with the electrolyte [32,33]. In addition, Hou et al. [33] reported that pyridinic-N boosts the electrochemical performances of the electrodes, which can be considered an essential advantage for ZnCl_2_-activated biochars.

To further evaluate the chemical composition of the biochar samples, Table 2 show the quantitative analyses for the C, O, and N and the I_D_/I_G_ ratio (based on Raman analysis). ZnCl_2_ biochar presented the highest C content, while the oxygen content was much higher in the KOH biochar (see Table 2). The nitrogen content is almost the same in both samples, while a residual Zn content is present in the biochar, which could positively affect the electrochemical performance of the material.

**Figure 5:**
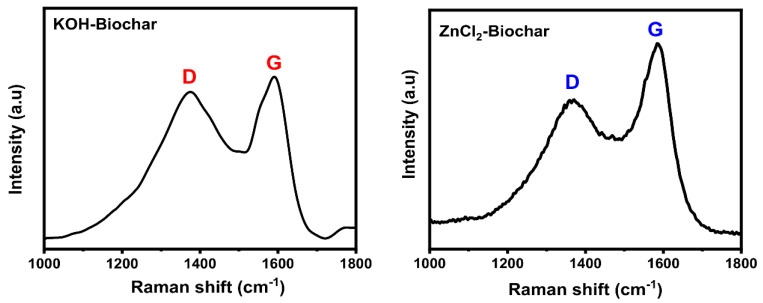
Raman spectra of biochar samples.

Raman spectroscopy is considered one of the most informative methods for evaluating the structural perfection and degree of order/graphitization of bio-based carbon materials [16,34]. Raman spectra of the biochars are shown in Figure 5. Both biochars exhibit two typical carbon characteristic diffraction peaks at 1370 cm^−1^ and 1590 cm^−1^, representing the D and G bands, respectively. The D band refers to the degree of chaos or imperfect structure in carbon materials, while the G peak corresponds to the ordered carbon structure [17].

According to Raman analysis, the intensity ratio of D band to G band (I_D_/I_G_) can be calculated, which is generally used to appraise the graphitization degree of carbon material [16,21,34,35,36] (see Table 2). It is well known that graphite has a high conductivity degree and is a very efficient material used as an electrode for ESS. Thus, the degree of graphitization is a valuable tool for understanding the carbon-based material’s suitability as electrode material [34,35,36]. The ZnCl_2_ and KOH biochars presented I_D_/I_G_ values of 0.94 and 0.60, respectively. The low I_D_/I_G_ value suggests that the material has closer to perfect and orderly graphite structures with a high graphitization degree; a high I_D_/I_G_ indicates that the material has more structural defects in its structure [16,21,34,35,36]. The biochar made via ZnCl_2_ activation presented a higher I_D_/I_G_ value (0.94) than KOH activation (0.60); therefore, KOH biochar had the highest graphitization degree. These results match SSA values because KOH biochar had the highest SSA, suggesting a lot more defects and disordered structure.

**Figure 6:**
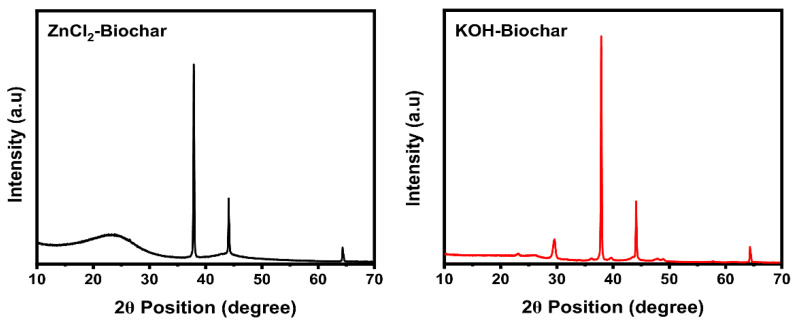
XRD patterns of biochar samples.

The XRD patterns of the biochar samples are shown in Figure 6. The patterns show important differences between both samples. It seems that the different chemical treatments affected the biochar structures.

The ZnCl_2_ biochar exhibited a typical diffraction pattern for amorphous biochars in 20–30° and three crystalline peaks in 38°, 44°, and 69° (which are assigned to the sample holder of aluminum). It is pointed out that the presence of amorphous material is required for carbon electrodes due to its network pores and vacancies [37]. For KOH-activated biochar, four evident crystalline peaks at 30°, 38°, 44°, and 69° (with the last three associated with the substrate) and additional and very small peaks at 23°, 36°, 39.5°, and 48.8° can be identified. 

The other crystalline peaks in both samples can be related to the high concentration of calcite in its structures. It is well known that spruce bark is biomass rich in calcium that, when is subjected to pyrolysis, reacts to form calcite [38].

Comparing the XRD of both biochars, it seems that KOH yielded a sample with more inorganic compounds such as calcite and quartz (electronic inert elements) [37,38], which can negatively affect the electrochemical properties of the electrode material.

**Figure 7:**
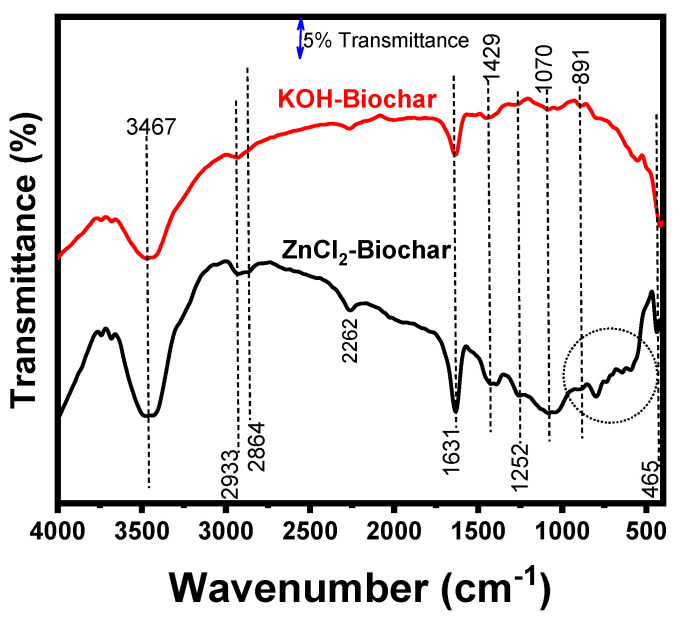
FTIR spectra of biochar samples.

FTIR spectra (see Figure 7) were performed to identify the presence of the functional groups on biochar samples. It is possible to observe that the different chemical treatments affected the chemical functionalities on the biochar surfaces. The band at 3467 cm^−1^ represents the O–H stretching vibration in carboxyl and phenol groups [10,11,12,15].

Two small peaks related to CH– stretching at 2933 and 2864 cm^−1^ were observed in ZnCl_2_ biochar, while only the first one was observed in KOH biochar. The peak at 2262 cm^−1^ can be attributed to hydrogen-bonding in OH, while in 1631 cm^−1^ it could be related to the asymmetric stretching of the O=C of carboxylates.

Small peaks at 1252, 792, and 646 cm^−1^ can be observed only in ZnCl_2_ biochar as well as others in the area highlighted by the circle inside the figure, suggesting that the ZnCl_2_ activation generated more functionalities on its surface. The presence of more functionalities on the ZnCl_2_ biochar surface can be related to the different activation mechanisms previously discussed. The existence of a high number of functional groups on biochar surfaces is often related to better electrochemical properties since it increases the electrode–electrolyte interactions [2,13,14].

### 3.3. Electrochemical Characterization

Electrochemical impedance spectroscopy (EIS) is a non-steady-state method that provides relevant information about several mechanisms at interfaces between the electrolyte and electrode [39]. In particular, the direct relationship between the time-dependent voltage (V) and current (I) defines the impedance (*Z* = *V*/*I*) that can be converted in terms of the complex capacitance *C* (with $j=\sqrt{-1})$

(1) $$C=C^{\prime }+jC^{\prime \prime }\;$$

From the definition of current I and capacitance *C* (*I* = d*Q*/d*t* and *C* = *Q*/*V*), both in terms of the charge *Q*, it is possible to write that

(2) $$I=\frac{V}{\left|Z\right|}=\frac{dQ}{dt}=jQ\omega \;\to \;\frac{\;Q}{V}=C=\frac{1}{j\omega \left|Z\right|}\;$$

With $\left|Z\right|=\sqrt{{Z^{\prime }}^{2}+{Z^{\prime \prime }}^{2}}$ . Equation (2) can be finally written in terms of the real and imaginary parts of the complex capacitance, as follows:

(3) $$C^{\prime }=\frac{-Z^{\prime \prime }}{2\pi f{\left|Z\right|}^{2}}$$

(4) $$C^{\prime \prime }=\frac{Z^{\prime }}{2\pi f{\left|Z\right|}^{2}}$$

where *C*′ is the capacitance at steady-state and *C*″ is related to the dielectric loss. In addition to the information provided by the complex capacitance, the frequency–response analysis (obtained from Nyquist plots) and response–time data (phase vs. frequency plot data) [40] are relevant information concerning diffusive properties, internal resistance, and resistive–capacitive transitions.

**Figure 8:**
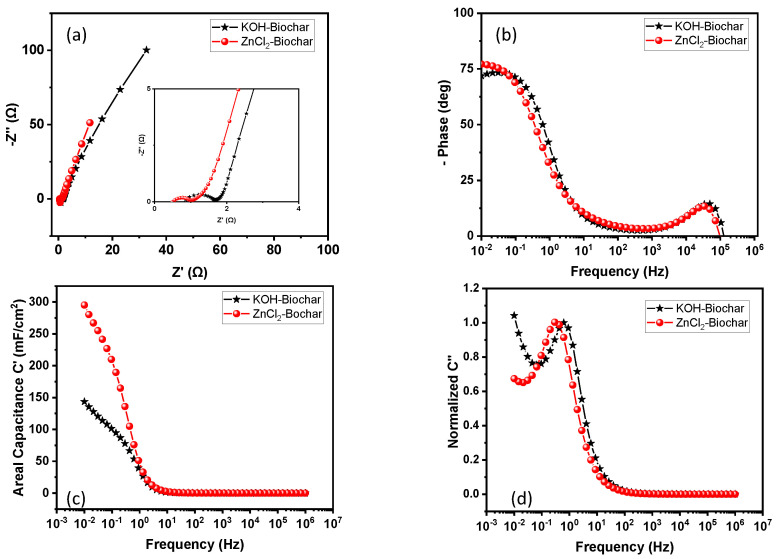
Electrochemical characterization curves for supercapacitors prepared with electrodes of KOH-based biochar (in black) and ZnCl_2_ biochar (in red): (**a**) Nyquist plots, (**b**) Bode plot of phase angle versus frequency, (**c**) real part of areal capacitance versus frequency, and (**d**) imaginary part of capacitance versus frequency.

Figure 8 summarize EIS-based curves, revealing the relevant electrochemical properties of the electrodes. Nyquist plots in Figure 8a are composed of a small semicircle at a higher frequency followed by a straight line at a low frequency. The capacitive behavior of samples can be explored from the relative variation in the linear line slope at a low frequency, while the intersection with the *x*-axis provides the estimative about the internal resistance. Results indicate lower internal resistance for ZnCl_2_ biochar (R = 0.55 Ω) against 0.86 Ω for KOH biochar. Regarding the slope in the low-frequency region, the inset of Figure 8a confirm a similar slope for both devices, indicating a minimal difference for diffusive transport along with the structures. Relative to the phase versus frequency dependence in the Bode plot (Figure 8b), an important parameter to be considered is the value of the phase angle that intercepts the y-axis. The values for ZnCl_2_- and KOH-based biochars are −77.13° and −71.93°, respectively, (the value of −90° is expected for ideal capacitors), indicating that ZnCl_2_-based samples obtain the best properties for a capacitor.

In addition, the typical behavior for the real part of capacitance was observed for both experimental systems with an increase from almost zero at higher frequencies to a maximum value at lower frequencies—characterizing a typical transition from resistive to capacitive behavior confirmed from the peak in the imaginary part of the capacitance. As shown in Figure 8c, confirming previous results from internal resistance and phase angle, the best performance in terms of the real part of capacitance at the quasi-stationary limit of frequency was observed for ZnCl_2_-based biochar with capacitance in order of 295 mF cm^−^^2^ in comparison with 143 mF cm^−^^2^ obtained for sample activated by KOH. The imaginary part of capacitance confirmed a breaking frequency established in the transition from resistive to capacitive behavior (values of 0.29 Hz and 0.62 Hz for ZnCl_2_ and KOH, respectively). The value of frequency at a maximum of *C*” was explored to estimate the time constant for charge/discharge—the calculated values were 3.44 s for ZnCl_2_-based biochar and 1.61 s for KOH-based biochar. 

Based on the previous results, it was possible to observe that the best performance is attributed to samples prepared via ZnCl_2_, as indicated by lower internal resistance and higher modulus of phase angle at low frequency, which justifies the good performance in terms of the areal capacitance.

**Figure 9:**
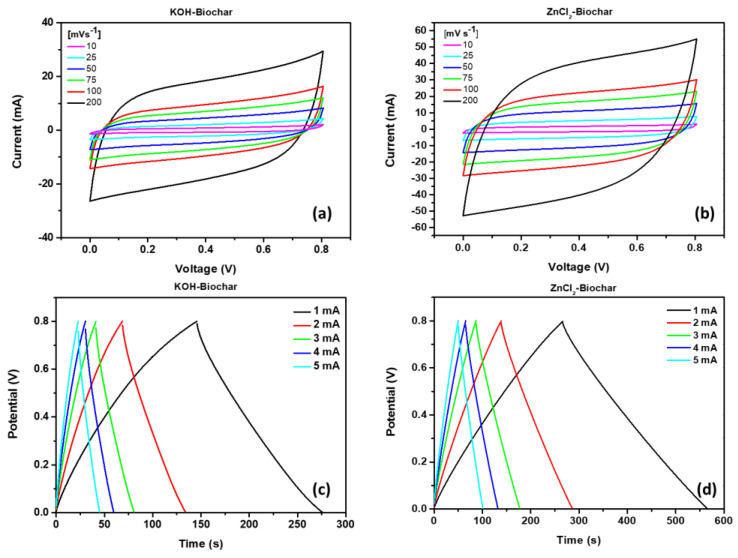
Cyclic voltammetry curves for ZnCl_2_-SC (**a**) and KOH-SC (**b**) at different scan rates (10 mVs^−1^ to 200 mVs^−1^) and galvanostatic charge–discharge curves for ZnCl_2_-SC (**c**) and KOH-SC (**d**) at a current of 1 mA to 5 mA.

Complementary and standard characterizations were evaluated (cyclic voltammogram, charge–discharge curves, and impedance spectrum), which returned data to calculate the areal capacitance, power density, and energy density for experimental systems. Cyclic voltammograms for ZnCl_2_-SC and KOH-SC are shown in Figure 9a,b, respectively. From these curves, it is possible to note that the corresponding current observed for ZnCl_2_-SC devices at the same scan rate is higher than the observed for KOH-SC devices. It introduces direct consequences on the area enclosed in the curve, affecting the calculus of the resulting capacitance. For both experimental systems, a quasi-rectangular behavior was observed at a low scan rate. At increasing values of the scan rates, an evident change can be seen in the curve format that acquires a prolate behavior with a cone-shaped response at a high limit (200 mVs^−1^). According to Liu et al. [41], the abundance of oxygenated functional groups (OH, C=O, COOH) may enhance the pseudocapacitive of biochar-based electrodes, which justifies the prolate behavior in CV curves.

The response of the devices at distinct current density was evaluated from galvanostatic charge–discharge, summarized in Figure 9c,d for samples ZnCl_2_-SC and KOH-SC, respectively. It is worth mentioning that the format and limiting values from these curves reveal important working mechanisms from respective devices [42,43,44]. Quasi-linear behavior was observed in the response of the supercapacitors, with a longer time interval for a complete charge–discharge cycle at lower current densities, as expected [42,43,44]. It is worth mentioning that a negligible IR drop was observed in the change from charge–discharge cycles, characterizing the low level of ohmic loss, an essential advantage in the overall energy storage process.

The reduced IR drop in both experimental systems can be confirmed from the low internal resistance of samples, as described in Figure 9a, with corresponding values of 0.55 Ω for ZnCl_2_-based and 0.86 Ω for KOH-based samples.

Data from CV and GCD curves were applied in Equations (5)–(7) to return values of areal capacitance (*C_ar_*) and the Ragone plot, calculated as follows:

(5) $$C_{ar}=\frac{2\;\times \;D_{ar}\;\times \;I}{V^{2}\;\times \;A}$$

(6) $$E_{D}=\frac{C_{ar}\times V^{2}}{2\;\times \;3600}$$

(7) $$P_{D}=\frac{3600\;\times \;E_{D}}{\Delta t}$$

From Equation (5), *D_ar_* is calculated from the area under the discharge curve, I is the current under the discharge process, *V* is the voltage window for the discharge curve, and *A* is the area of the device. The energy density (*E_D_*), given by Equation (6), is calculated from areal capacitance and the voltage window, while the power density (*P_D_*) considers the resulting energy density and the total time involved in the device’s discharge.

**Figure 10:**
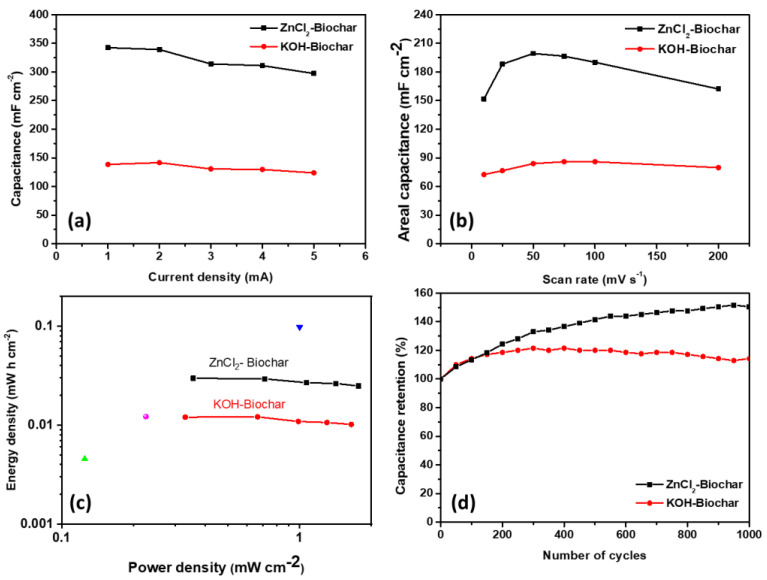
(**a**) Dependence of areal capacitance with the current for different samples, (**b**) areal capacitance calculated from the area under CV curves and (**c**) Ragone plot for samples ZnCl_2_−SC and KOH−SC and comparison with data reported in the literature (green-up triangle—Ref. [45], purple circle—Ref. [46] and blue down triangle—Ref. [47]), (**d**) capacitive retention for ZnCl_2_−SC and KOH−SC at GCD curves acquired at a current of 5 mA.

Based on Equations (5)–(7) and the data in Figure 9, curves of areal capacitance and Ragone plot were obtained. As shown in Figure 10a, the areal capacitance for samples ZnCl_2_-SC returned a better performance than the corresponding KOH-SC, with higher values for capacitance for all of the current densities. In the same direction, the areal capacitance calculated from the area under the CV curves confirmed that the best performance in the capacitance was observed for ZnCl_2_-based biochars for all scan rates (Figure 10b). The superior electrochemical performance was confirmed from the Ragone plot (see Figure 10c), in which it was possible to observe higher values for both energy density and power density for ZnCl_2_-based samples in comparison with corresponding KOH-based electrodes. In addition, points with different colors were introduced in Figure 10c to compare these values with those reported in the literature for corresponding experimental systems. As can be seen, our samples present good power density and competitive energy density in comparison with data in the literature.

Degradation assays were evaluated for both experimental systems, in successive GCD assays in which a current of 5 mA was applied in symmetric supercapacitors (KOH-SC and ZnCl_2_-SC) with results summarized in Figure 10d. As can be seen, due to the prevailing contribution of carbon derivatives in both systems, a high retention degree in the capacitance was observed for ZnCl_2_-SC and KOH with increasing retention degree (negligible degradation) after 1000 cycles of charge/discharge at a high current density. Denmark observed a similar effect in supercapacitors based on molasses-based co-doped carbon with KOH as the electrolyte [42]. In the literature, this process is attributed to the delayed activation of the electrochemical properties of the device, since the electrochemical charge–discharge reactions progressively activated mechanisms in extra non-accessible sites being favored by the increasing wettability at the electrode/electrolyte interface (higher activation degree is observed for sample ZnCl_2_—as expected). These data confirm the high stability degree of the device in a basic electrolyte.

The electrochemical characterization strongly indicated that the bark biochars exhibited satisfactory supercapacitor application performance. Although the nature of each carbon electrode is different, and each one of them has its advantages and drawbacks, here, we provided comparison data with other published works. Table 3 show the areal capacitance values for our bark biochar electrodes compared with other reported electrodes in the literature. 

According to Gou et al. [43], it is challenging to produce biomass carbon anodes for supercapacitors with high areal capacitance because many biomass precursors provide carbon anodes with areal capacitance lesser than 28 mF cm^−2^. Therefore, developing economically feasible carbon materials with high areal capacitance is imperative to meet the demand of future applications in energy storage.

By Table 3, which compares the areal capacitance of some electrode material, it is possible to see that the areal capacitance for ZnCl_2_ biochar is the second-highest among the listed electrodes in Table 3, showing to be very competitive biochar for employment in supercapacitors and perhaps in other electrochemical systems. ZnCl_2_ biochar displayed an areal capacitance of 342 mF cm^−2^ at 5 A g^−1^, while some graphene materials presented much less areal capacitance [44,45,46]. It is worth highlighting that the production costs for graphene are incredibly higher when compared to that of ZnCl_2_ biochar. Consequently, if the production cost is added to the desirable properties, ZnCl_2_-activated spruce biochar could be classified as an excellent electrode material for supercapacitors with high areal capacitance.

Therefore, the valorization of spruce bark as a sustainable and green strategy to obtain porous and efficient anodes materials for high-performance supercapacitors represents a remarkable example of constructing highly sustainable energy storage devices.

The best performance of ZnCl_2_-based SCs in comparison with KOH-based can be considered as a conjunction of the several factors, described as follows:

(i) The measured residual zinc in samples of ZnCl_2_ Biochar, even at a small quantity, might influence the capacitance of the electrodes since Zn metal has a high theoretical capacity (820 mAh g^−1^) [51], which is more than double the theoretical capacity of graphite (372 mAh g^−1^);
(ii) The morphology of ZnCl_2_-based biochars with a rich distribution of cavities and holes are driven forces that facilitate the permeation of electrolytes along with the electrodes, contributing to a more effective process of charge separation.

In addition to these aspects, the low internal resistance of material associated with the higher phase angle measured of ZnCl_2_-based biochar contributes to the overall best performance in areal capacitances and energy storage. It is a consequence of the higher mesoporosity of ZnCl_2_ that facilitates the electrolyte permeation and charge accumulation in assembled supercapacitors.

## 4. Conclusions

The chemical activation of biochars for carbon derivatives is a critical step in developing more effective electrodes for supercapacitors. As commonly used activators, the electrochemical properties of biochars based on ZnCl_2_ and KOH were evaluated, and the data was compared with the microstructure of the resulting materials. The higher mesoporosity degree was observed for samples activated by ZnCl_2_ while widening micropore was obtained with KOH activation. Consequently, better capacitive properties associated with lower internal resistance were obtained for ZnCl_2_-based biochar that returned an areal capacitance in order of 342 mF cm^−^² in comparison with 138.49 mF cm^−^² for KOH-based biochar. The doping process with a residual zinc element and higher hydrophilic behavior for ZnCl_2_-based samples can be considered positive aspects that improve the performance of the mesopore-type supercapacitors. The results obtained in this work strongly suggest that the spruce bark can be considered a high-efficiency precursor for biobased electrode preparation to be employed in SCs.

## Figures and Tables

**Table 1:** Textural properties of the biochars.

Parameters.	ZnCl_2_ Biochar	KOH Biochar
SSA (m^2^ g^−1^)	1018	2209
Mesopore surface area (m^2^ g^−1^)	456	449
Mesopore surface area (%)	46.0	22.6
Micropore area (m^2^ g^−1^)	562	1710
Micropore area (%)	54.0	77.4
Total pore volume (cm^3^ g^−1^)	0.78	1.50
Micropore volume (cm^3^ g^−1^)	0.41	0.25
Mesopore volume (cm^3^ g^−1^)	0.37	1.25
Average pore size (nm)	2.21	2.70

**Table 2:** XPS elemental composition of the biochar samples.

		XPS			I_D_/I_G_	HI
Samples	C 1s	O 1s	N 1s	Zn 2p		
ZnCl_2_ biochar	93.3	4.7	1.5	0.5	0.94	0.90
KOH biochar	86.6	10.5	1.7	-	1.16	0.96

**Table 3:** Comparison of areal capacitances of porous carbon-based supercapacitors prepared (with different types of electrodes) reported in the literature and devices prepared in this work.

Electrode Material (Red for Biochar-Based Materials)	Areal Capacitance	Electrolyte	Current Density (A g^−1^) or Scan Rate (mV s^−1^)	Reference
Wheat straw cellulosic biochar	0.3 mF cm^−2^	6 M KOH	0.5 A g^−1^	[43]
Graphene fiber	3.3 mF cm^−2^	PVA/H_3_PO_4_		[44]
Modified Graphene fiber with polyaniline	66.6 mF cm^−2^	PVA/H_3_PO_4_		[44]
Graphene oxide-conductive polymer fiber	131 mF cm^−2^	PVA/H_3_PO_4_		[45]
Graphene modified with polyaniline	87.8 mF cm^−2^	EMITFSI/PVDF-HFP	0.22 mA cm^−2^	[46]
N-doped porous carbon fiber sheets from biomass-flax	703 mF cm^−2^	6 M KOH	20 mA cm^−2^	[47]
Lignin-carbon decorated with molybdenum disulfide	16 mF cm^−2^	6 M KOH	10 mV s^−1^	[48]
Lignocellulose-derived phosphorus-doped carbon	146 mF cm^−2^	6 M KOH	10 A g^−1^	[49]
Sputtered carbon-doped titanium nitride	45.81 mF cm^−2^	6 M KOH	10 mV s^−1^	[50]
KOH biochar	138.49 mF cm^−2^	5 M KOH	5 A g^−1^	This work
ZnCl_2_ biochar	342 mF cm^−2^	5 M KOH	5 A g^−1^	This work

## Data Availability

Not applicable.
