# Correction: Lima et al. Facile Synthesis of Sustainable Biomass-Derived Porous Biochars as Promising Electrode Materials for High-Performance Supercapacitor Applications. *Nanomaterials* 2022, *12*, 866

**DOI:** 10.3390/nano15231794

**Published:** 2025-11-28

**Authors:** Ravi Moreno Araujo Pinheiro Lima, Glaydson Simões dos Reis, Mikael Thyrel, Jose Jarib Alcaraz-Espinoza, Sylvia H. Larsson, Helinando Pequeno de Oliveira

**Affiliations:** 1 Institute of Materials Science, Federal University of Sao Francisco Valley, Petrolina 56304-205, Brazil; raviplima.engmec@gmail.com (R.M.A.P.L.); helinando.oliveira@univasf.edu.br (H.P.d.O.); 2 Department of Forest Biomaterials and Technology, Swedish University of Agricultural Sciences, Biomass Technology Centre, SE-90183 Umeå, Sweden; mikael.thyrel@slu.se (M.T.); sylvia.larsson@slu.se (S.H.L.); 3 Departamento de Química, Universidad Autónoma Metropolitana, Iztapalapa, Mexico City 09340, Mexico; josejarib@gmail.com

In the original publication [1], there were several mistakes made as published. In Table, the porosity data for ZnCl_2_ biochar are 1018 m^2^ g^−1^, 456 m^2^ g^−1^ and 562 m^2^ g^−1^ for specific surface area, mesopore area, and micropore area, respectively. The value of I_D_/I_G_ for KOH biochar is 0.60, as stated in ref. [2], and the correct Raman spectroscopy is depicted as BC6. It is worth mentioning that the samples used in the publication [1] were defined from previous studies on the optimization of preparation conditions of carbon derivatives. Therefore, some characterization data were previously published in Refs. [2,3].

In ref. [2], fifteen carbon-based materials were also prepared according to the *Box–Behnken design*, but KOH was used as a chemical activator. Sample BC6 (pyrolyzed at 900 °C for 2 h and at a ratio of 1:1, biomass: KOH) was denoted KOH biochar in our publication [1].

In ref. [3], fifteen different carbon-based materials were prepared, employing statistical analysis to study the influence of the synthesis methods on physicochemical properties and their ability to be used as adsorbents for drug removal. As a result, sample AC10 (pyrolyzed at 900 °C for 2 h and at a ratio of 1:1, biomass: ZnCl_2_) was chosen and denoted ZnCl2 biochar in ref. [1]. Corrected Table 1 can be found below:

Also, in the original publication [1], there was a mistake while fitting the XPS spectra, published as Figure 4. The mistake was made by the corresponding author while interpreting the XPS spectra, in which the fitting of the raw spectra was not properly performed. The inconsistency in the figure has been corrected and the updated figure appears below.

**Figure 4:**
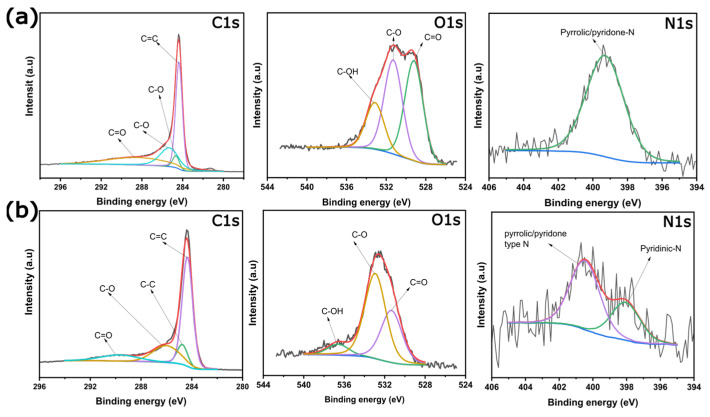
Carbon, oxygen, and nitrogen XPS spectra for (**a**) KOH biochar and (**b**) ZnCl_2_ biochar.

A correction has been made to 3.2. Chemical and Functional Characterization of the Biochars, Paragraphs 2 and 3: New text:

The asymmetric C 1s spectra could be deconvoluted into four peaks as highlighted in Figure 4. These peaks correspond to C=C, C–C, C–O–C, and C=O bonds, typically of activated carbons [16,25,27].

O1s spectra exhibited some differences in their peak intensities regarding the chemical activation process. Both biochars present O1s spectra deconvoluted to three chemical oxygen states corresponding to (i) oxygen double-bonded with carbon (C=O) in carbonyl and quinone-like structures, (ii) oxygen singly bonded to carbon (C-O) in aromatic rings, in phenols and ethers, and (iii) hydroxyl groups (-OH). The presence of this oxygen species can improve the hydrophilicity degree of the sample, which can reflect in a better interaction between the solid and liquid phase (electrode–electrolyte), characterizing an advantage for permeation of aqueous electrolyte ions into the biochar-based electrode structure.

A correction has been made to 3.2. Chemical and Functional Characterization of the Biochars, Paragraph 7, Line 6: New text:

The ZnCl_2_ and KOH biochars presented I_D_/I_G_ values of 0.94 and 0.60, respectively. The low I_D_/I_G_ value suggests that the material has closer to perfect and orderly graphite structures with a high graphitization degree; a high I_D_/I_G_ indicates that the material has more structural defects in its structure [16,21,34–36]. The biochar made via ZnCl_2_ activation presented a higher I_D_/I_G_ value (0.94) than KOH activation (0.60); therefore, KOH biochar had the highest graphitization degree.

The authors state that the scientific conclusions are unaffected. This correction was approved by the Academic Editor. The original publication has also been updated.

## Figures and Tables

**Table 1:** Textural properties of the biochars.

Parameters.	ZnCl_2_ Biochar	KOH Biochar
SSA (m^2^ g^−1^)	1018	2209
Mesopore surface area (m^2^ g^−1^)	456	449
Mesopore surface area (%)	46.0	22.6
Micropore area (m^2^ g^−1^)	562	1710
Micropore area (%)	54.0	77.4
Total pore volume (cm^3^ g^−1^)	0.78	1.50
Micropore volume (cm^3^ g^−1^)	0.41	0.25
Mesopore volume (cm^3^ g^−1^)	0.37	1.25
Average pore size (nm)	2.21	2.70
